# American Medical Association

**Published:** 1878-05-20

**Authors:** 


### AMERICAN MEDICAL ASSOCIATION.

The American Medical Association will hold its 29th annual session at Buffalo, N. Y., commencing June 4th next, 11 o'clock a. m.

The State, county and district societies throughout the Union, of legitimate bodies, have the privilege of sending one delegate for every ten of its resident members, and one for every additional fraction of more than half that number.

It is desired that medical societies forward a list of their delegates as soon as possible.

A large attendance is expected, and the meeting will probably be one of unusual interest.

